# Tuning magnetocrystalline anisotropy by cobalt alloying in hexagonal Fe_3_Ge^1^

**DOI:** 10.1038/s41598-018-32577-x

**Published:** 2018-09-21

**Authors:** Michael A. McGuire, K. V. Shanavas, Michael S. Kesler, David S. Parker

**Affiliations:** Oak Ridge National Laboratory, Oak Ridge, Tennessee 37831 USA

## Abstract

We show using both experimental and theoretical methods that cobalt substitution in the hexagonal ferromagnet Fe_3_Ge suppresses the planar magnetic anisotropy and favors a uniaxial state. Uniaxial ferromagnetism is observed at room temperature for cobalt concentrations of only a few percent, and 10% substitution fully suppresses the planar magnetic structure at least down to 5 K, with only a small effect on the magnetization and Curie temperature. First principles calculations predict strong uniaxial magnetocrystalline anisotropy and promising permanent magnet properties for higher cobalt concentrations. Although these high Co concentrations were not realized experimentally, this work suggests that the rare-earth-free Fe_3_Ge structure supports intrinsic magnetic properties that may enable promising permanent magnet performance.

## Introduction

Research targeting new permanent magnet materials is driven by both technological and economic factors. Magnets that perform better at elevated temperatures while tolerating strong demagnetizing fields are required for improving electric motors and generators for transportation and energy applications, while magnets comprising abundant elements are required to keep costs low and ensure supply stability^1^. Finding materials that meet both these requirements is a challenging task^2–4^.

A good permanent magnet must have a high Curie temperature, a large magnetization, and a substantial coercive field to resist demagnetization. The coercivity is generally limited by the anisotropy field, the field required to rotate the moment from the easy to the hard direction, which is typically large only in the case of uniaxial magnetocrystalline anisotropy. For reference, hexagonal Ba-ferrite (BaFe_12_O_19_) has a Curie temperature *T*_*C*_ = 740 K, saturation magnetization *J*_*S*_ = 0.48 T, and anisotropy constant *K*_1_ = 0.33 MJ/m^3^, while the corresponding values for tetragonal Nd_2_Fe_14_B are 588 K, 1.6 T, and 4.3 MJ/m^3^ ^5^. Large magnetic moments and high Curie temperatures are associated with 3*d* transition metals, and the magnetocrystalline anisotropy is closely related to the crystallographic symmetry; in particular, cubic compounds generally exhibit weak magnetic anisotropy. Thus, a common strategy for identifying potential permanent magnet materials is to examine compounds, like those above, with high concentrations of iron in hexagonal or tetragonal crystal structures.

**Figure 1:**
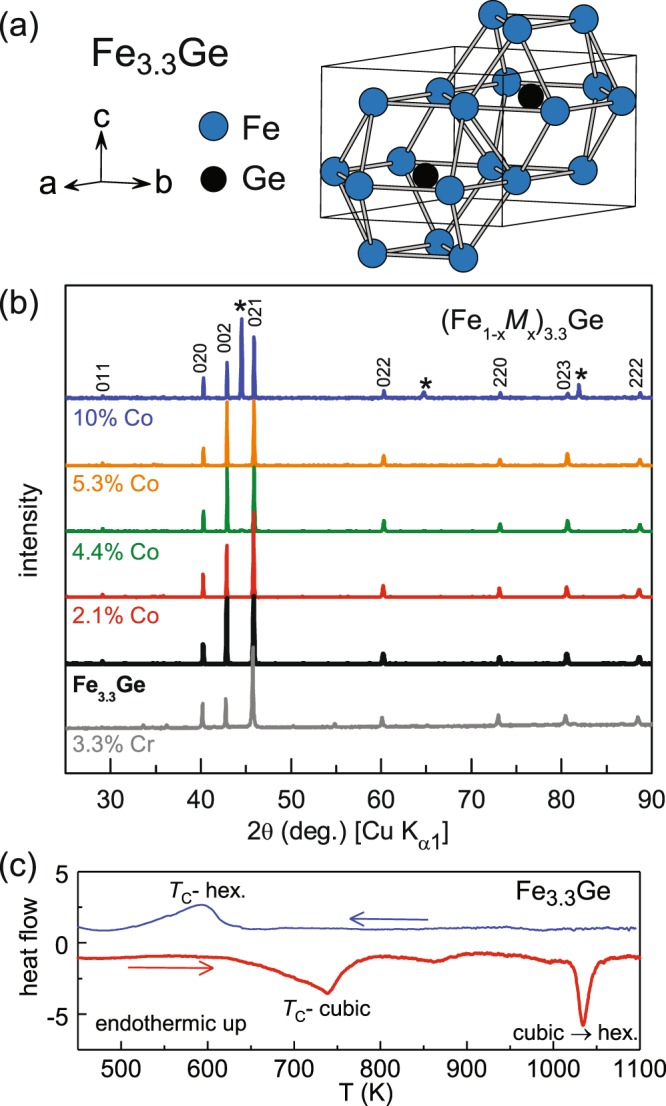
(**a**) Structure of hexagonal Fe_3.3_Ge showing the face-sharing, Ge-centered anticuboctahedra formed by the Fe atoms. (**b**) Powder x-ray diffraction patterns from Co and Cr substituted Fe_3.3_Ge samples after quenching from 1173 K, labeled by their compositions. Reflections from the hexagonal Fe_3.3_Ge structure are marked with indices, and asterisks mark reflection from the cubic polymorph present in the 10% Co sample. (**c**) Thermal analysis data from a cubic Fe_3.3_Ge sample (20 K/min).

^1^

The intermetallic compound Fe_3_Ge is an iron rich ferromagnet that forms in both a cubic (Cu_3_Au-type, *Pm*$\mathop{3}\limits^{-}$*m*) and a hexagonal (Mg_3_Cd-type, *P*6_3_/*mmc*)) structure^6,7^. According to the published phase diagram, the cubic structure is stable from 673 to 973 K, while the hexagonal structure is stable from about 973 to 1395 K^8^. Both phases can be stabilized at room temperature by quenching^9^. Cubic and hexagonal Fe_3_Ge both have high magnetizations, with moments of about 2 *μ*_*B*_/Fe, and high Curie temperatures of about 750 and 650 K, respectively^6,9^. This, along with its crystallographic anisotropy, makes the hexagonal phase of substantial interest as a permanent magnet material. Its structure is shown in Fig. 1a. At room temperature the magnetocrystalline anisotropy is planar, so that the ordered moments lie in the *ab*-plane, but a spin reorientation occurs near *T*_*SR*_ = 380 K and the anisotropy is uniaxial at higher temperature with the moments along the hexagonal *c*-axis^10^. The anisotropy field is expected to be small in planar magnetic structures, so extending the stability of the uniaxial state to lower temperature is desirable. In general, realizing such control over magnetocrystalline anisotropy is key in developing potential new magnets.

We have recently shown that substitution of Si for Ge in Fe_3_Ge has the effect of extending the stability range of the uniaxial ferromagnetism, suppressing *T*_*SR*_ by about 70 K (to 312 K) and *T*_*C*_ by about 30 K (to 595 K), with little effect on the saturation magnetization^11^. This is consistent with predictions from first principles calculations, which indicate uniaxial behavior could be realized over the whole temperature range for high Si concentrations; however, the solubility was found to be limited in experiments to about 6%. There have also been studies of transition metal substitutions. Albertini *et al*. examined a sample with 20% of the Fe replaced with Mn, and found that indeed *T*_*SR*_ was reduced to 126 K, well below room temperature^12^. Unfortunately, *T*_*C*_ was reduced to 493 K and the magnetization was reduced by 19%. Nickel substitution was studied by Kanematsu and Takahashi, and the hexagonal phase was stabilized for up to 10% Ni, with a reduction in magnetic moment but little dependence of the Curie temperature on Ni concentration^13^. No reports of cobalt substitution in Fe_3_Ge were located in the literature, perhaps because no compounds with composition Co_3_*X* (*X* = Si, Ge, Sn) are known to form^13^.

Here we investigate experimentally and theoretically the effects of alloying cobalt into hexagonal Fe_3_Ge. Preliminary experimental results for Cr substitution are also presented. We find that Co rapidly suppresses the spin reorientation temperature with a relatively small suppression of the Curie temperature and magnetization. We observe uniaxial ferromagnetism at room temperature with only a few percent Co substitution, and complete suppression of the planar phase near 10% Co. First principles calculations confirm this trend, and predict the potential for good permanent magnet properties if the hexagonal phase can be stabilized for higher cobalt concentrations.

## Results and Discussion

While the binary phase diagram shows hexagonal Fe_3_Ge stable for 24–25 at.% Ge^8^, in our previous study^11^ we found the hexagonal phase formed best with 23 at.% Ge corresponding to Fe_3.3_Ge. Polycrystalline pellets of nominal compositions (Fe_1−*x*_*M*_*x*_)_3.3_Ge were made and characterized as described in the Methods section. Powder x-ray diffraction (PXRD) patterns for the samples described in this paper are shown in Fig. 1b. The hexagonal phase was formed when heated at 1173 K for Fe_3.3_Ge, *M* = Co samples with nominal values of *x* = 0, 0.25, 0.05, 0.07, as well as a Cr substituted sample with nominal composition (Fe_0.90_Cr_0.10_)_3.3_Ge. A small amount, about 2% of the cubic phase was seen in the nominal *x* = 0.07 sample prepared this way (see Supplemental Information). Heating the 0.10 Co sample to this temperature produced a 1:1 mixture of hexagonal and cubic phases. Further heat treatments of this composition tended to increase the cubic content. An additional sample was made with Co content of *x* = 0.15, and it showed only the cubic phase after heating at temperatures ranging from 1073 to 1223 K. Grinding the samples was found to severely degrade the crystallinity of these metastable materials (as seen by powder x-ray diffraction), so diffraction measurements were taken from as-fired pellet surfaces. While the focus of the present work in on the hexagonal phase, one sample of Fe_3.3_Ge was heated at 848 K to convert it to the cubic phase, and then used for differential thermal analysis (Fig. 1c). Upon heating, the Curie temperature (*T*_*C*_) of the cubic phase is observed followed by the transformation to the hexagonal structure (near 1030 K). The *T*_*C*_ of the hexagonal phase is seen upon cooling.

Energy dispersive x-ray spectroscopy (EDS) was used to determine the chemical compositions of the main phase in the pellets. The results, along with lattice parameters determined from the PXRD patterns, are collected in Table 1. All samples contained 23 at.% Ge, consistent with the 3.3:1 metal to germanium ratio. The measured cobalt concentrations are close to the nominal values, but the Cr content in the nominally 10% Cr sample was only about 3%, suggesting good solubility for Co and poor solubility for Cr in Fe_3.3_Ge. The apparent poor solubility of Cr indicates that much of the loaded Cr segregates into secondary phases. These phases were not identified in the present study, but a small amount of Cr_2_O_3_ was seen in the powder x-ray diffraction data from the surface of the as-fired pellet (additional peaks in the 3.3% Cr data in Fig. 1b, see also Supplementary Information). Cr substitution is seen to expand the hexagonal lattice, while Co substitution produces a contraction. In the following, the EDS-determined composition will be used to refer to the different samples.

**Table 1:** Chemical and structural information for the (Fe_1−*x*_*M*_*x*_)_3.3_Ge samples used in this study.

*M*	*x*_nom_	*x*_EDS_	at.% Ge	*a* (Å)	*c* (Å)
—	0	0	23 (1)	5.1759(1)	4.2217(1)
Co	0.025	0.021 (2)	23 (1)	5.1767(1)	4.2219(1)
Co	0.05	0.044 (4)	23 (1)	5.1729(1)	4.2172(1)
Co	0.07	0.053 (3)	22.5 (5)	5.1729(1)	4.2186(1)
Co^*a*^	0.1	0.10 (1)	23 (1)	5.1700(2)	4.2158(2)
Cr	0.1	0.033 (6)	22.8 (8)	5.1810(2)	4.2265(2)

^*a*^contains about 50% cubic phase with *a* = 5.7581(2) Å.

**Figure 2:**
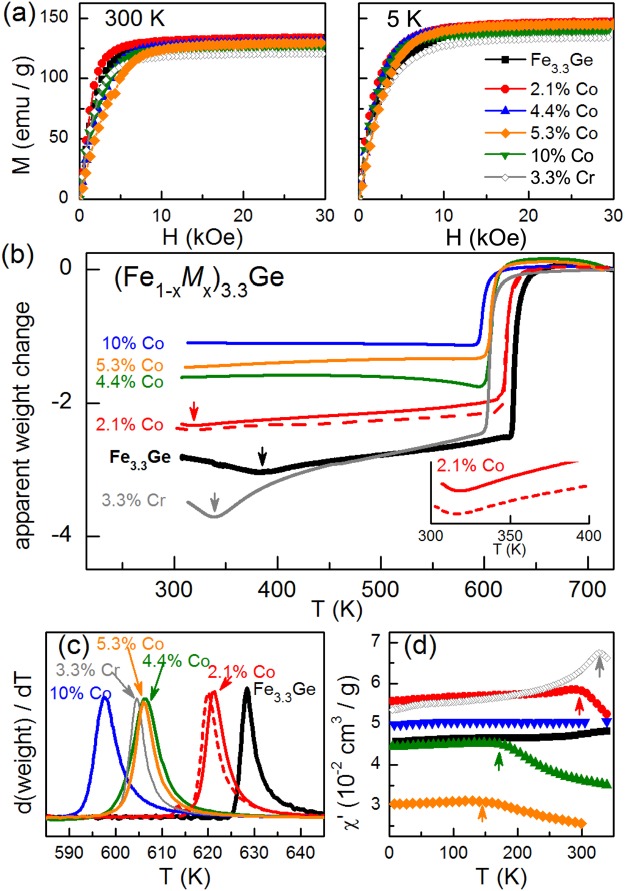
Magnetic behavior of (Fe_1−*x*_*M*_*x*_)_3.3_Ge. (**a**) Magnetization vs applied magnetic field measured at 300 and 5 K. (**b**) Thermomagnetic analysis showing a sharp change in apparent weight (magnetic force) at *T*_C_ and a minimum at *T*_SR_ (indicated by arrows). (**c**) Temperature derivative of the apparent weight used to determine *T*_C_. (**d**) ac magnetic susceptibility used to determine *T*_SR_ (indicated by arrows).

The magnetic properties of the (Fe_1−*x*_*M*_*x*_)_3.3_Ge samples were investigated using dc and ac magnetization measurements and thermogravimetric analysis (TGA) conducted in a small magnetic field gradient. The results are shown in Fig. 2. Isothermal magnetization curves (Fig. 2a) measured at 300 and 5 K show ferromagnetic responses in all samples. The saturation moments are collected in Table 2 and a slight decrease is observed with increasing Co concentration. Magnetic transition temperatures were determined from the TGA data at high temperature (Fig. 2b,c) and ac susceptibility data near and below room temperature (Fig. 2d). The Curie temperature is identified by a sharp change in the apparent weight of the sample, which includes a contribution from the magnetic force between the sample and the permanent magnet. The temperature derivative of this signal is used to define *T*_*C*_. The spin reorientation, from planar at low temperature to axial at high temperature, is observed as a local minimum in the apparent weight where the powder averaged magnetic susceptibility is highest (anisotropy is smallest). These points are marked by arrows in Fig. 2b. Data from two different 2.1% Co samples are shown. Near and below room temperature, ac magnetization measurements were used to locate the local maximum in susceptibility corresponding to the spin reorientation (Fig. 2d). Reasonably good agreement is observed between *T*_*SR*_ values determined by the two techniques for (Fe_0.97_Cr_0.03_)_3.3_Ge and (Fe_0.98_Co_0.02_)_3.3_Ge (Table 2).

**Table 2:** Measured magnetic properties of (Fe_1−*x*_*M*_*x*_)_3.3_Ge samples.

Compound	*T*_*C*_ (K)	*T*_*SR*_ (K)	*M*_*S*_ (emu/g)	*M*_*S*_ (*μ*_*B*_/F.U.) *T* = 5 K	*J*_*S*_ (T)	*M*_*S*_ (emu/g)	*M*_*S*_ (*μ*_*B*_/F.U.) *T* = 300 K	*J*_*S*_ (T)
Fe_3.3_Ge	628	383^*a*^	146	6.7	1.49	134	6.1	1.36
(Fe_0.98_Co_0.02_)_3.3_Ge	621	315^*a*^, 290^*b*^	147	6.8	1.50	134	6.2	1.36
(Fe_0.96_Co_0.04_)_3.3_Ge	606	170^*b*^	144	6.6	1.47	130	6.0	1.32
(Fe_0.95_Co_0.05_)_3.3_Ge	606	120^*b*^	145	6.7	1.48	129	5.9	1.31
(Fe_0.90_Co_0.10_)_3.3_Ge	598	not obs.	139	6.4	1.41	127	5.9	1.29
(Fe_0.97_Cr_0.03_)_3.3_Ge	605	339^*a*^, 325^*b*^	134	6.2	1.36	121	5.5	1.23

^*a*^Determined from thermomagnetic analysis. ^*b*^Determined from magnetic susceptibility.

The data in Table 2 shows that the Curie temperature is suppressed by cobalt substitution at a rate of about -3 K per at.% Co. However, *T*_*SR*_ changes by about −50 K per at.% Co. The spin-reorientation temperature is suppressed below room temperature for Co concentrations exceeding about 2%, and below 5 K for 10% Co. This means that Co-substituted Fe_3.3_Ge is a uniaxial ferromagnet from room temperature up to *T*_*C*_ with only a few percent Co. Chromium substitution appears to reduce *T*_*C*_ at a higher rate with a weaker effect on *T*_*SR*_. The spin reorientation in (Fe_0.97_Cr_0.03_)_3.3_Ge occurs near 330 K; planar ferromagnetism persists at room temperature in this alloy.

**Figure 3:**
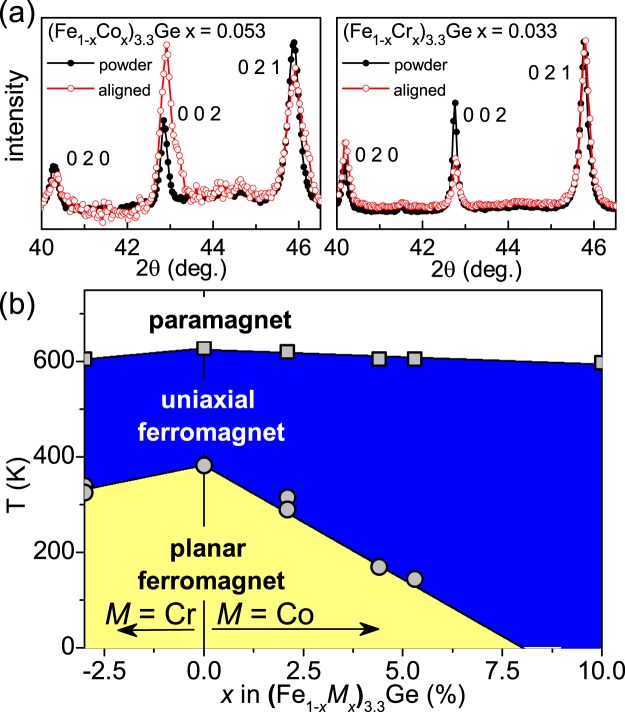
(**a**) Powder x-ray diffraction patterns comparing results from magnetically aligned particles (aligned) with results from conventional measurements without alignment (powder). (**b**) Phase diagram for Cr- and Co-susbtituted hexagonal Fe_3.3_Ge showing the composition dependence of *T*_C_ (squares) and *T*_SR_ (circles).

The magnetic anisotropy was confirmed by diffraction. For these measurements, samples of hexagonal (Fe_0.95_Co_0.05_)_3.3_Ge and (Fe_0.97_Cr_0.03_)_3.3_Ge were ground into powders. The grinding was done by hand, and was relatively gentle, to preserve the hexagonal phase and its crystallinity as much as possible while producing relatively small particles. The powders were mounted on vacuum grease on a substrate with a strong permanent magnet underneath producing a field perpendicular to the substrate. This results in preference for the surface normal to be aligned with the easy axis (uniaxial anisotropy) or in the easy plane (planar anisotropy), enhancing or suppressing the 00L reflections, respectively. Figure 3a shows sections of diffraction patterns containing 020, 002, and 021 reflections. For the (Fe_0.95_Co_0.05_)_3.3_Ge powder the aligned sample has enhanced 002 intensity, indicating uniaxial ferromagnetism at room temperature, while the aligned (Fe_0.97_Cr_0.03_)_3.3_Ge sample has suppressed 002 intensity, consistent with planar ferromagnetism at room temperature. With these behaviors confirmed, the phase diagram shown in Fig. 3b can be constructed from the experimental results in Fig. 2 and Table 2.

The effect of cobalt substitution in Fe_3_Ge was also examined using first principles calculations. Results are shown in Table 3. Structures with 0, 10, 20, and 30% Co substituted for Fe in Mg_3_Cd-type Fe_3_Ge were examined using the virtual crystal approximation (VCA). The results in Table 3 show that Co reduces the net moment, as seen in the experimental data. The calculated magnetizations for Fe_3_Ge and (Fe_0.9_Co_0.1_)_3_Ge are close to the experimental values for Fe_3.3_Ge and (Fe_0.90_Co_0.10_)_3.3_Ge (Table 2), and furthermore the anisotropy constant *K*_1_ is calculated to be −0.34 MJ/m^3^ for Fe_3_Ge. This negative sign indicates planar anisotropy, and the good agreement with experiment suggests the reliability of this spin-polarized density functional theory for this materials class. As shown in Table 3, cobalt substitution increases the magnetic anisotropy K_1_ value and a uniaxial ground state (positive *K*_1_) is predicted to emerge at approximately 10% Co. These findings are in good agreement with the experimental results presented above.

**Table 3:** Calculated magnetic anisotropy constant *K*_1_ and magnetic polarization *J* of (Fe_1−*x*_Co_*x*_)_3_Ge.

Compound	*K*_1_ (MJ/m^3^)	*J* (T)
Fe_3_Ge	−0.38	1.49
(Fe_0.9_Co_0.1_)_3_Ge	0.03*	1.47
(Fe_0.8_Co_0.2_)_3_Ge	0.63	1.42
(Fe_0.7_Co_0.3_)_3_Ge	1.23	1.35

*This value is within the accuracy of the calculations.

Crucially, a significant positive *K*_1_ is calculated for larger Co concentrations. For 30% Co, an anisotropy field of *H*_*A*_ = 2*K*_1_/*J*_*S*_ = 1.8 MA/m or *μ*_0_*H*_*A*_ = 2.2 T can be estimated. The calculated moment and anisotropy are sufficiently large that it is plausible that a suitably microstructured sample of such a material could achieve significant coercivity - an important step towards realization of a permanent magnet. Note that the theoretical upper limit on energy product *BH*_*max*_ is ${{\rm{J}}}_{S}^{2}/(4{\mu }_{0})$, or approximately 360 kJ/m^3^ (45 MG-Oe) for the 30% Cobalt sample, if one could here achieve the required minimum coercivity of *J*_*S*_/2, or 0.68 T. This is approximately 30% of the anisotropy field H_*A*_, which falls at the upper bound of typically achievable coercivities. Hence it is possible that a suitably prepared sample could obtain large fractions of the *BH*_*max*_ figure quoted above. This is comparable to present values for the Nd_2_Fe_14_B magnet, however, we note that this is an optimistic upper bound based on what is presently a hypothetical compound with the hexagonal Fe_3_Ge structure. Most importantly, the calculations indicate that strong uniaxial anisotropy may be achievable in this structure type without the rare-earth elements often considered indispensable for the generation of magnetic anisotropy. This is consistent with several recent works finding substantial magnetic anisotropy in rare-earth-free magnetic materials, for example Fe_3_Sn, HfMnP and Mn_3_Si_2_Te_6_^14–16^, and should warrant future theoretical and experimental studies of these and similar transition metal compounds.

As is well known, calculation of K_1_ is an involved endeavor and requires great care to achieve accurate results, due to the need to isolate energy differences of often less than 1 meV per unit cell from a total energy which may be ten or more orders of magnitude larger. Indeed, it is only in recent decades, due to great increases in computing power, that it has become computationally possible to generate reliable results for K_1_. Our procedure for accurate calculation of K_1_ is as follows: first, we performed a careful relaxation of the internal coordinates to a fairly small force threshhold (3 mRyd/Bohr). Next, we chose a very large planewave basis set, parametrized by our choice of an *RK*_*max*_ of 9.0, where *RK*_*max*_ is the product of the smallest LAPW sphere radius and the largest plane-wave wavevector. For reference, we note that (although not used here) for magnetic systems with comparatively small atoms, such as B or C, a smaller *RK*_*max*_ may be used as the effective *RK*_*max*_ for the magnetic atoms such as Fe or Co will be larger due to the generally larger sphere radius of these atoms. The next step in the procedure is to check convergence with respect to the number *n*_*k*_ of *k*-points in the Brillouin zone. We have done this for the 20% Co case, for *n*_*k*_ at values of 1, 5, 10 and 30 × 10^3^ in the full Brillouin zone and present the results in Table 4. As is evident, the change in *K*_1_ between the last two steps is only a few percent, so we consider that for the *K*_1_ behavior of interest here 10,000 *k*-points are sufficient and we have used this throughout this work.

**Table 4:** *K*-point convergence check of K_1_ for (Fe_0.8_Co_0.2_)_3_Ge.

K-points (×10^3^)	*K*_1_ (MJ/m^3^)	Percent difference from final result
1	0.624	−1.7
5	0.561	−11.6
10	0.614	−3.2
30	0.634	—

The calculations were run to self-consistency with the energy difference between the last two convergence cycles generally well less than 10^−6^ Ryd, or about 0.03 MJ/m^3^ in K_1_. All of the calculated K_1_ magnitudes, with the exception of the 10% Co case are much larger than this energy difference and accordingly we attach a high degree of reliability to these values. With regards to the value for the 10% Co case (Table 2) of 0.03 MJ/m^3^, this value is effectively within the present accuracy of the calculation and therefore not distinguishable from zero, as noted in the Table. However, the significant variation in K_1_ from the pure case to the 20% Co case is evidence that, computationally speaking, the 10% Co case is rather near the point at which axial behavior begins, consistent with our experimental finding.

To address potential concerns about the accuracy of the VCA in this system, we have also conducted calculations of the magnetization and magnetic anisotropy of an ordered Fe_2_CoGe cell, again with the LDA. Calculations of magnetic anisotropy on such a cell are often problematic due to the need to maintain the crystal symmetry (in this case hexagonal) in order to obtain an accurate value. However, the use of a spin-orbit calculation effectively splits the sixfold Fe 6 *h* site into a fourfold and twofold Fe site, and we have substituted Co onto the twofold Fe site. Nearest-neighbor distances between these two Co are 3.58 Å, significantly larger than the Fe-Fe nearest and next-nearest neighbor distances of 2.50 and 2.59 Å in the base compound, so that we may consider this an appropriate structural model for a random alloy.

Note that the use of the “two-fold” nomenclature, while technically accurate due to the lowering of crystalline symmetry by planar magnetic moment orientation in this spin-orbit calculation, should not be mistaken, energetically speaking, for a true *distinct* two-fold crystallographic site. The energy difference between planar and c-axis moment orientations in this system (the magnetic anisotropy) is no more than 0.5 meV per unit cell here, while the binding energy associated with the placement of Fe (or Co) on its site, while not calculated here, is likely in the one to several eV range. The use of this site (for Co substitution) is designed to achieve two purposes essential here for the calculation of K_1_: the maintenance of hexagonal symmetry, or equivalence of the a and b directions; and the avoidance of unphysical (i.e. too large or small distances between the Co atoms) structures creating a spurious anisotropy. The pattern assumed here meets both of these criteria.

The saturation spin moment of this Fe_2_CoGe cell was 10.97 *μ*_*B*_/unit cell, or about 4% smaller than the value for the VCA Fe_2.1_Co_0.9_Ge cell (note the slight difference in stoichiometry). This level of agreement is fairly good, considering that the Co spin moment is roughly half that of the Fe, and suggests the accuracy and applicability of the VCA here. Similarly, this ordered Fe_2_CoGe cell is calculated to exhibit axial magnetic anisotropy, as in the VCA cell, with a K_1_ value of 1.15 MJ/m^3^, in good agreement with the VCA result of 1.23 MJ/m^3^. Accordingly, we consider that the VCA results here are generally rather accurate, and that therefore there is potential for significant magnetic anisotropy in these Fe_3−*x*_Co_*x*_Ge alloys, if the stability issue can be overcome.

## Conclusions

To summarize, we have demonstrated, both from experiment and theory, chemical control of the magnetic anisotropy in the Co-substituted Fe_3_Ge-based alloy system. In particular, first principles calculations predict that Co-substitution changes the magnetocrystalline anisotropy of the ground state from planar to uniaxial. This trend is reflected in the experimental results; the spin-reorientation temperature is suppressed to below room temperature with only a few percent cobalt and to below 5 K for 10% Co. Although the optimal Co concentrations predicted by theory could not be realized experimentally, our results suggest that Fe_3_Ge-related materials have the potential for good permanent magnet performance, and motivate further study of these systems, particularly in light of the ongoing search for “gap magnets”^3^.

## Methods

Polycrystalline pellets of nominal compositions (Fe_1−*x*_*M*_*x*_)_3.3_Ge were made by first arc-melting alloys of compositions *M*_3.3*x*_Ge and then grinding them together with iron powder according to the target stoichiometries. Fe_3.3_Ge was made from Fe and Ge powders. After mixing thoroughly, the powder samples were pressed into 12 mm diameter pellets and sealed inside fused silica tubes that had been evacuated and back-filled with about 1/4 atm ultra-high-purity argon. The ampoules were heated in resistive box furnaces and quenched in ice water.

Powder x-ray diffraction patterns were obtained using a PANalytical X’Pert MPD with Cu K _*α*1_ radiation. Semi-quantitative chemical analysis with energy dispersive x-ray spectroscopy was performed with a Hitachi T3000 SEM with Bruker Quantax 70 x-ray detector. Thermogravimetric and differential thermal analysis measurements were made with a Perkin Elmer Pyris TGA/DTA. To sense magnetic transitions in the TGA a small magnetic field gradient at the sample was produced by a permanent magnet affixed to the top of a sample space. The dc and ac magnetization data were collected using a PPMS and MPMS (Quantum Design).

Density functional theory calculations were performed using the all-electron linearized augmented planewave (LAPW) density functional theory code WIEN2K^17^, within the local density approximation. Interestingly, the generalized gradient approximation does not reproduce the experimentally observed transition from planar to axial behavior with Co alloying; with this functional the material remains planar in the alloying range studied here. The reasons for this are unclear; it is possible that the magnetism here contains a degree of itinerant character, for which the LDA would likely give a more accurate description. Sphere radii of 2.26 and 2.28 Bohr for Ge and Fe were used, along with an *RK*_*max*_ (the product of the smallest sphere radius and largest planewave expansion vector) of 9.0. Sufficient number of *k*-points - 5,000 or more in the full Brillouin zone - were used to ensure convergence. Internal coordinates were relaxed until residual forces were less than 3 mRyd/Bohr.

## Electronic supplementary material

Powder x-ray diffraction results

## Data Availability

The datasets generated during and/or analyzed during the current study are available from the corresponding author on reasonable request.

## Footnotes

1 Notice: This manuscript has been authored by UT-Battelle, LLC under Contract No. DE-AC05-00OR22725 with the U.S. Department of Energy. The United States Government retains and the publisher, by accepting the article for publication, acknowledges that the United States Government retains a non-exclusive, paid-up, irrevocable, world-wide license to publish or reproduce the published form of this manuscript, or allow others to do so, for United States Government purposes. The Department of Energy will provide public access to these results of federally sponsored research in accordance with the DOE Public Access Plan (http://energy.gov/downloads/doe-public-access-plan).
