# Supplementary material for: Tuning magnetocrystalline anisotropy by cobalt alloying in hexagonal Fe3Ge1
Source: Sci Rep. 2018 Sep 21;8:14206. doi: 10.1038/s41598-018-32577-x (PMC6155063; doi:10.1038/s41598-018-32577-x)
Supplement: Supplementary file 1 — Powder x-ray diffraction results [file 41598_2018_32577_MOESM1_ESM.pdf]

Supplementary Information:

Tuning magnetocrystalline anisotropy by cobalt alloying in hexagonal Fe_3Ge

Michael A. McGuire, K.V. Shanavas, Michael S. Kesler, and David S. Parker

Oak Ridge National Laboratory, Oak Ridge, Tennessee 37831 USA

Powder x-ray diffraction patterns showing Rietveld refinement fits for all samples with refined weight fractions for the identified phases listed and difference curves plotted below each pattern.

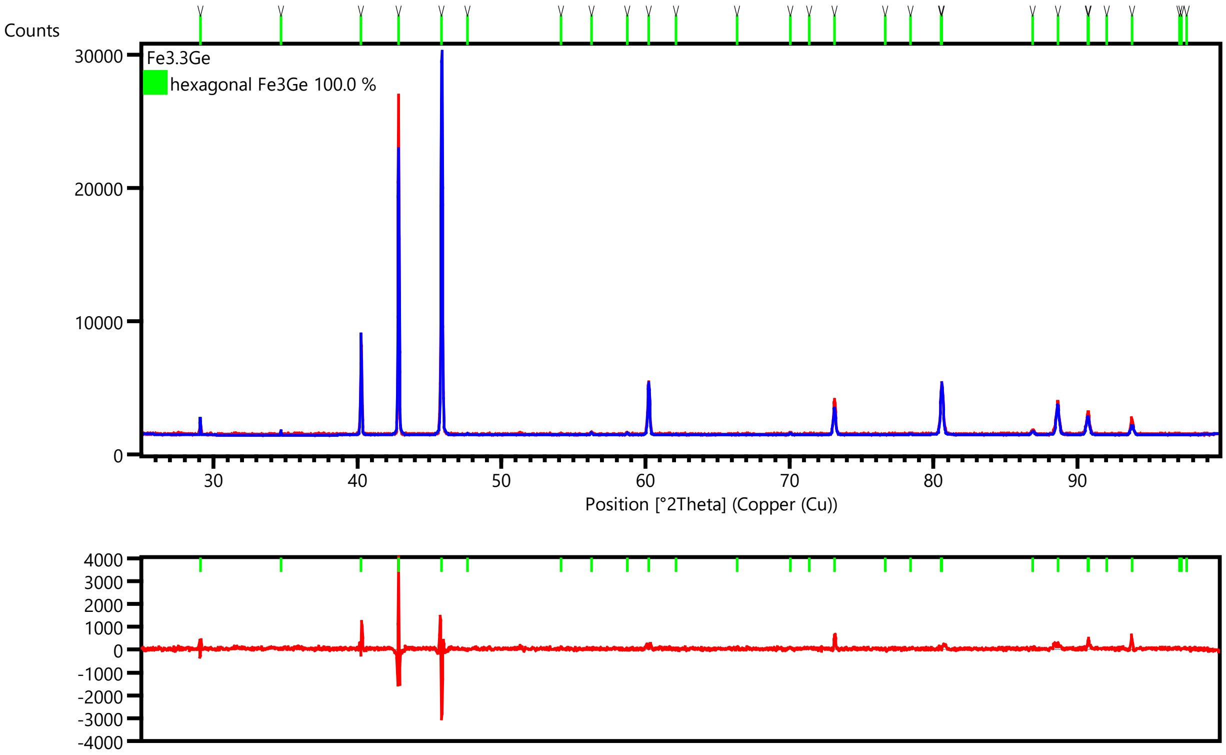

Figure S1. Rietveld fit to the $Fe_{3.3}Ge$ sample using the hexagonal Mg_3Cd structure.

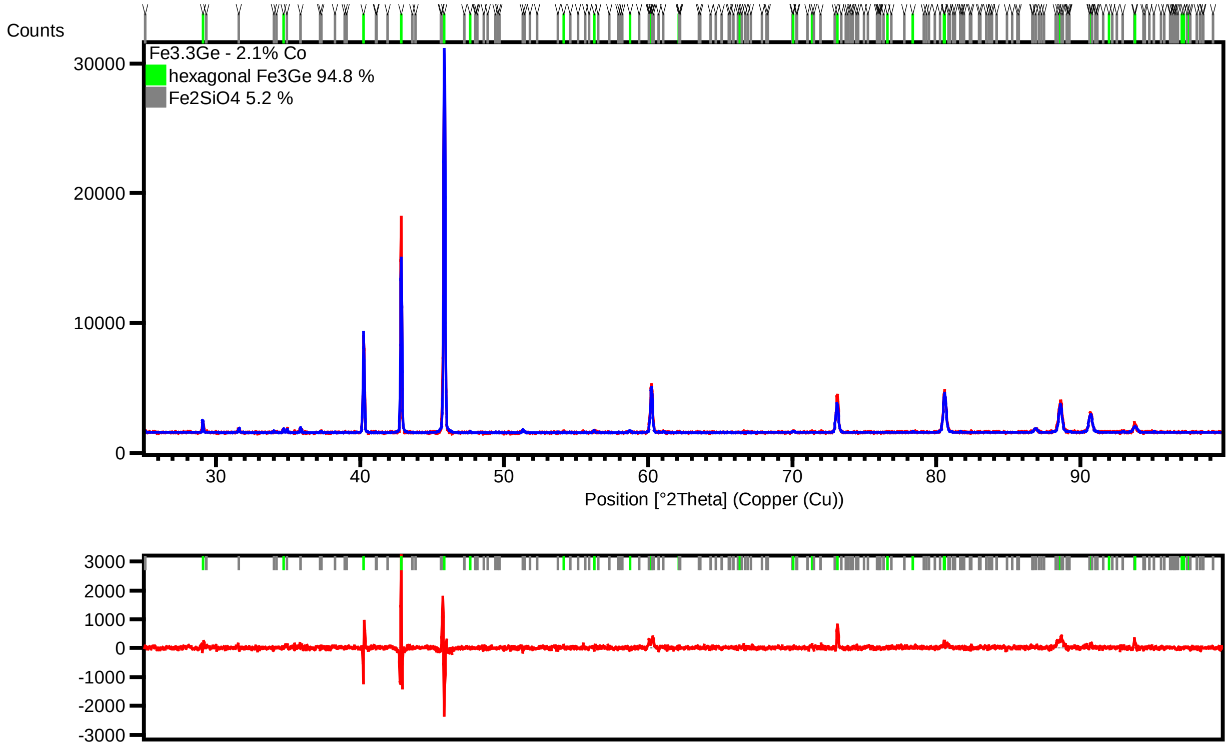

Figure S2. Rietveld fit to the $(Fe_{1-x}Co_x)_{3.3}Ge$ x = 0.021 sample using the hexagonal Mg_3Cd structure and Fayalite type Fe_2SiO_4.

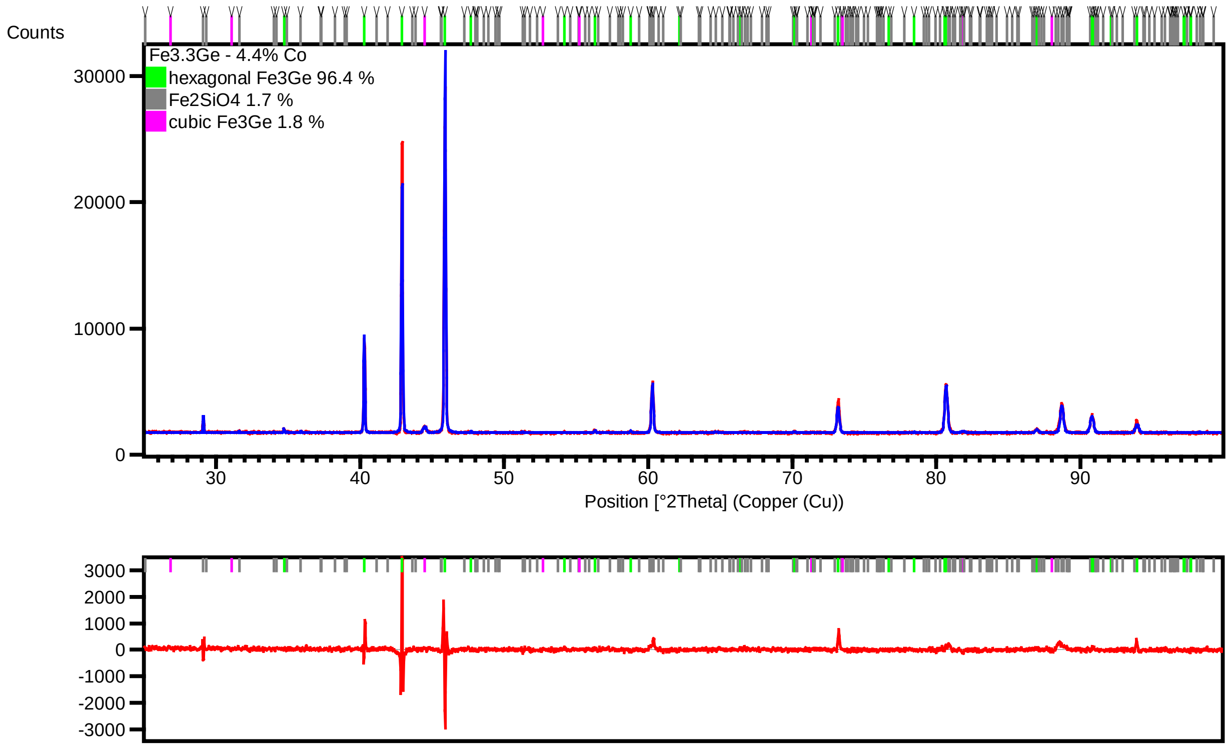

Figure S3. Rietveld fit to the $(Fe_{1-x}Co_x)_{3.3}Ge$ x = 0.044 sample using the hexagonal Mg_3Cd structure, the cubic Cu_3Au structure, and Fayalite type Fe_2SiO_4.

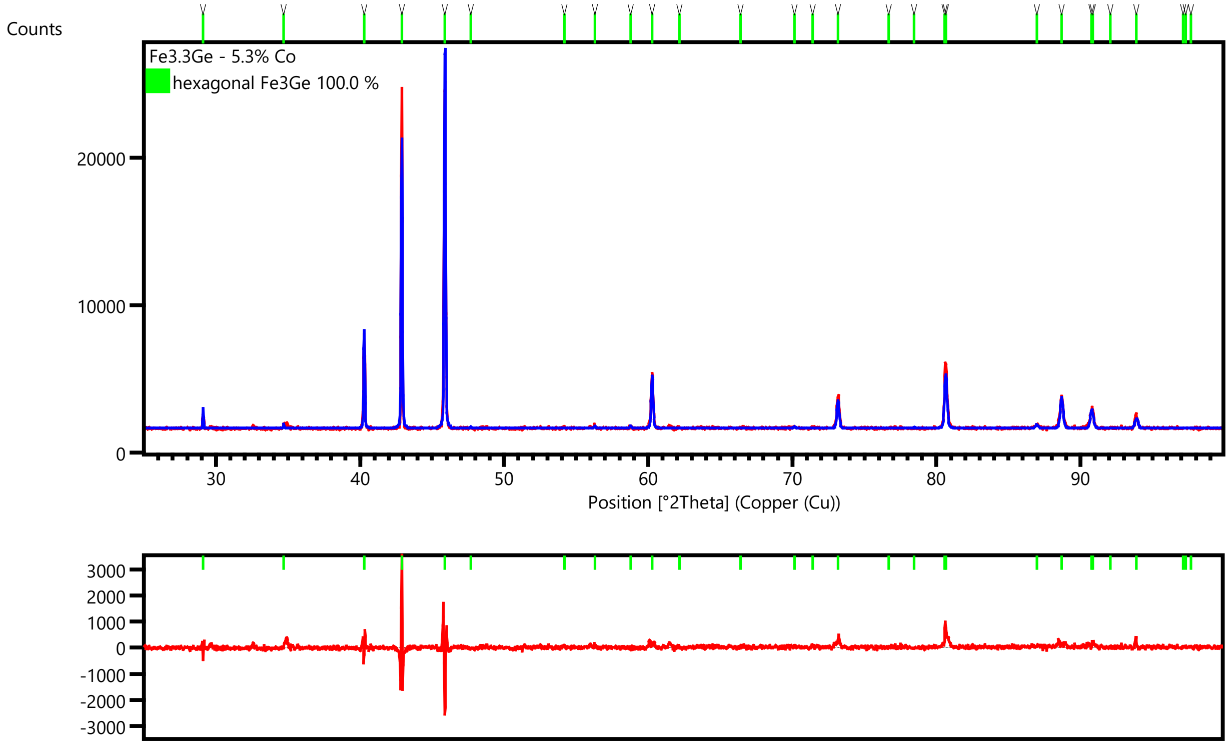

Figure S4. Rietveld fit to the $(Fe_{1-x}Co_x)_{3.3}Ge$ x = 0.053 sample using the hexagonal Mg_3Cd structure.

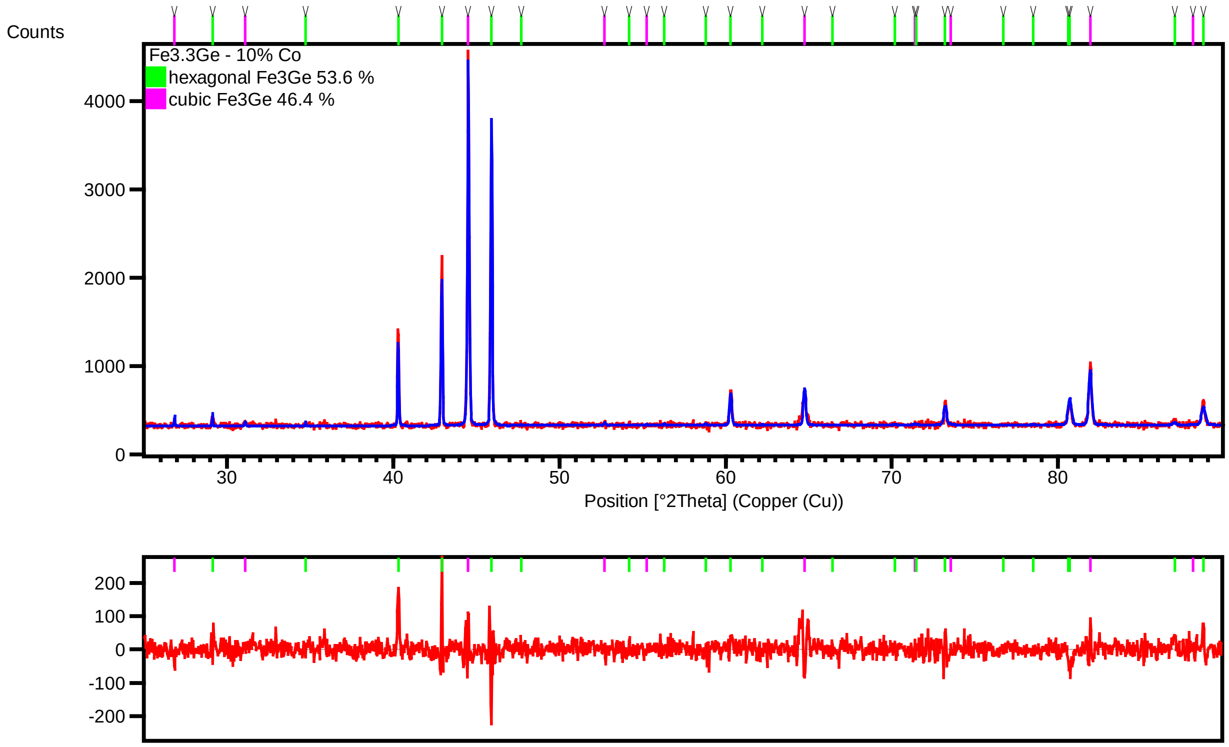

Figure S5. Rietveld fit to the $(Fe_{1-x}Co_x)_{3.3}Ge$ x = 0.10 sample using the hexagonal Mg_3Cd structure and the cubic Cu_3Au structure.

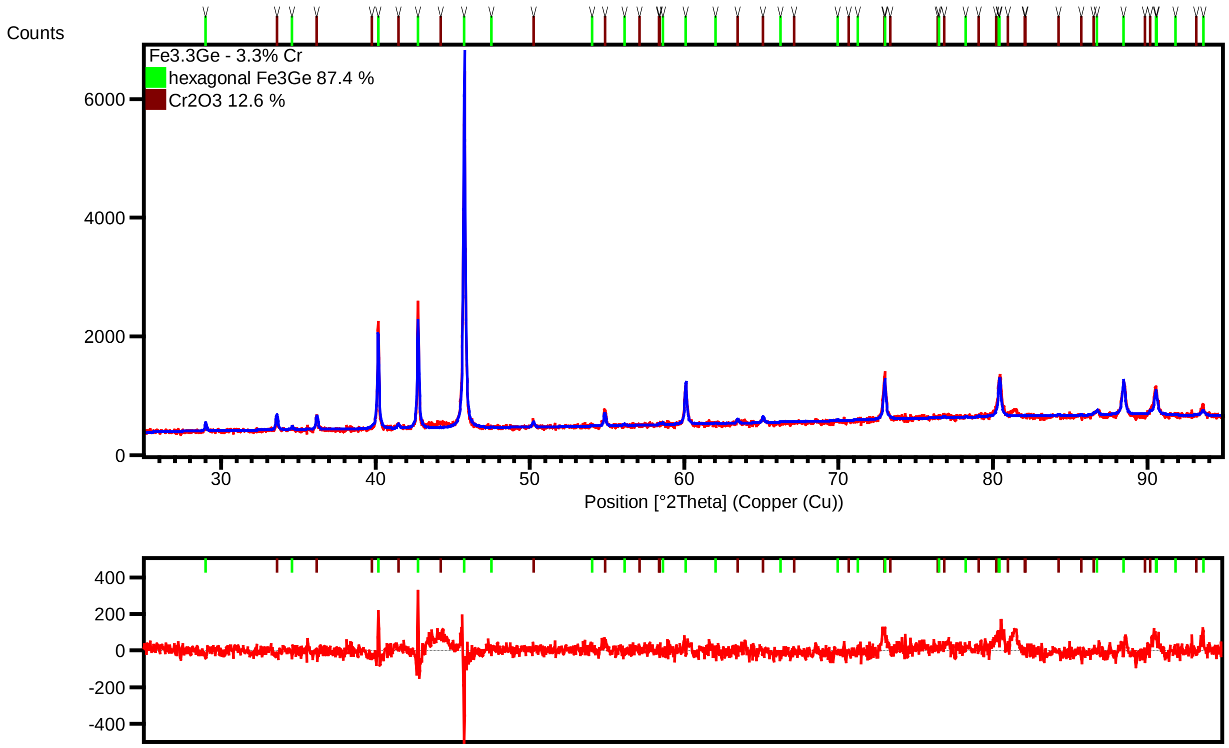

Figure S6. Rietveld fit to the $(Fe_{1-x}Cr_x)_{3.3}Ge$ x = 0.036 sample using the hexagonal Mg_3Cd structure and Eskolaite type Cr_2O_3.
